# 胸腺恶性肿瘤化疗的相关定义和策略

**DOI:** 10.3779/j.issn.1009-3419.2014.02.09

**Published:** 2014-02-20

**Authors:** Nicolas Girard, Rohit Lal, Heather Wakelee, Gregory J. Riely, Patrick J. Loehrer, 北京大学肿瘤医院 付浩

**Affiliations:** 1 Department of Respiratory Medicine, Louis Pradel Hospital Hospices Civils de Lyon, Lyon (Bron), France; 2 Cancer Services, Guy's and St. Thomas' Cancer Centre, London, United Kingdom; 3 Division of Oncology, depart-ment of Medicine, Stanford University, Stanford, California; 4 Thoracic Oncology Service, Memorial Sloan Kettering Hospital, New York, New York; 5 Division of Hematology and Oncology, Indiana University Melvin and Bren Simon Cancer Center, Indianapolis, Indiana

胸腺恶性肿瘤是少见的上皮源性肿瘤，但部分肿瘤侵袭性强且治疗效果不佳^[1]^。胸腺瘤多发于前纵隔，手术切除是主要的根治性治疗方式^[1]^。然而，30%的患者确诊时即为进展期胸腺瘤，包括侵犯邻近脏器，向胸膜、心包播散，以及胸腔外脏器的转移。对于进展期胸腺瘤，化疗有两个明确目的，其一是降低肿瘤负荷为后续手术或放疗创造机会，其二是延长疾病的控制时间。对于术后复发可以采取相同的化疗策略。尽管胸腺癌发病率很低，但诊断时多已是晚期，全身性治疗显得尤为重要。

虽然已有前瞻性临床试验，但胸腺肿瘤化疗仍建立在回顾性研究的基础上^[2-21]^。这些研究表明胸腺瘤对某些细胞毒性化疗药物较为敏感，但胸腺癌的敏感性较之胸腺瘤为差。然而，研究中患者的详细信息（如疾病范围或一般状况）记录不全，治疗的程序也模糊不清，难以比较。因此，有必要建立国际胸腺肿瘤协作组构建胸腺肿瘤前瞻性数据库，建立统一、规范的定义和标准。ITMIG已经对生存、复发评估终点以及疗效评估的方法进行了定义，但关于化疗的问题，包括治疗顺序、一般方案和类固醇激素的作用等尚未作出统一标准。为此，ITMIG首先由一个核心工作组回顾分析现有文献资料，并对胸腺瘤化疗的定义提出初步建议，由包括放射肿瘤专家在内的一个扩大工作组再次进行讨论。最终由所有ITMIG会员讨论并于2010年11月16日批准。本文就此作一综述。

## 1 胸腺肿瘤的化疗

### 1.1 治疗目的

化疗可用于胸腺肿瘤治疗的不同阶段（表 1）。对于局部进展期肿瘤，化疗可以是根治性治疗的组成部分，治疗目的是获得长期无瘤生存。因此对于局部进展期肿瘤，常采用化疗联合局部治疗的模式，如术前化疗+手术、手术+术后化疗或放化疗。对于进展期或已发生远处转移的胸腺瘤患者，化疗是姑息性治疗手段，目的是改善肿瘤相关症状，虽然有理由希望延长肿瘤控制时间，但并不期望根治肿瘤，此时，化疗就是单一的治疗手段。

**1:** 胸腺恶性肿瘤化疗相关报告指南 Summary of reporting guidelines and data fields for thymic malignancies treated with chemotherapy

化疗策略	
治疗目的	
根治性目的	
初始化疗	局部治疗前化疗——手术或放疗
	需记录治疗目的，如术前化疗或放疗前化疗
	需记录所有患者接受的最终治疗方案，如术前化疗或初始放化疗
术后化疗	应说明手术切除情况，如R0、R1、R2切除
姑息性目的	
姑息化疗	对未计划行手术或放疗的患者行单纯化疗
复发后化疗	经根治性治疗后肿瘤复发的化疗
	复发后根治性治疗，如术前、放化疗前或术后化疗，或复发后姑息性治疗，如单纯化疗
	需记录治疗目的和最终治疗方案
化疗报告指南	
方案	用药方案
	化疗周期
	药物剂量：>或 < 70%计划剂量
分析	胸腺瘤和胸腺癌治疗结果应分别评估
毒性	应根据NCI不良反应分级标准，报告3级-5级或药物限制性化疗毒性反应
	应报道急慢性毒性反应。需特别注意慢性化疗毒性反应，如心脏毒性
反应	肿瘤治疗反应的评估另文论述
	行奥曲肽治疗的患者需报道奥曲肽扫描结果
	记录抗肿瘤治疗对副瘤综合征的疗效
	应记录皮质醇激素治疗的药物剂量和治疗时间
复发	对于治疗停止15个月以内出现肿瘤生长，局部复发应与反跳性胸腺增生相鉴别
注：本表已获得版权所有者© 2011 by the International Association for the Study of Lung Cancer许可。	

因化疗目的不同化疗的反应率和生存率也有所不同，因此建议在化疗开始前首先明确治疗目的：①化疗目的是根治性或姑息性？②对以根治性为目的的化疗，接下来拟行手术或放疗或二者联合？然而最终治疗目的的确认需要根据肿瘤反应和其他指标进行调整，研究者应以化疗患者最终接受的治疗方案记录治疗目的，包含以上信息的前瞻性数据库可以使不同中心的报告有效的整合。由于初始化疗后行手术治疗和放疗的两组患者数量差异较大（表 2），提示这些研究的入组标准也不一致。这就要求我们记录统一的入组标准、治疗目的和最终给予的治疗方案。

**2:** 局部进展期胸腺恶性肿瘤的初始化疗或放化疗 Primary chemotherapy or chemoradiation for locally advanced thymic malignancies

研究	化疗方案	例数	肿瘤			研究类型	后续治疗（%）				
			累计周期	组织分类	分期		反应率（%）	手术	完全缓解	放疗	姑息化疗
化疗											
Macchiarini^[14]^	CEE	7	2	T/TC	Ⅲ	Phase Ⅱ	100	100	57	0	0
Berruti^[16]^	ADOC	6	2	T	Ⅲ-Ⅳa	Phase Ⅱ	83	?	17	?	?
Rea^[6]^	ADOC	16	6	T	Ⅲ-Ⅳa	Retrospective	100	100	69	0	0
Venuta^[3]^	CEE	15	14	T/TC	Ⅲ	Retrospective	66	100	?	?	?
Bretti^[7]^	ADOC/PE	25	11	T/TC	Ⅲ-Ⅳa	Retrospective	72	68	44	?	?
Kim^[15]^	CAPP	22	10	T		Phase Ⅱ	77	100	72	0	0
Lucchi^[4]^	CEE	36	27	T/TC	Ⅲ-Ⅳa	Retrospective	67	69	78	19	3
Jacot^[5]^	CAP	5	6	T/TC	Ⅲ-Ⅳa	Retrospective	75	38	25	50	12
Yokoi^[8]^	CAMP	14	15	T/TC	Ⅲ，Ⅳa-b	Retrospective	93	64	14	14	21
Kunitoh^[17]^	CODE	21	8	T	Ⅲ	PhaseⅡ	62	62	43	24	14
放化疗											
Loehrer^[18]^	CAP	23	12	T/TC	Ⅲ-Ⅳa	Phase Ⅱ	70	15	0	70	15
Berruti^[19]^	ADOC	16	7	T	Ⅲ-Ⅳa	Phase Ⅱ	81	56	56	31	13
Wright^[9]^	PE, ADOCCAP, CEE	10	9	T/TC	Ⅲ-Ⅳa	Retrospective	40	100	80	0	0
CAP，ADOC和PE方案见表 1。CODE方案：顺铂（25 mg/m^2^/wk），长春新碱（1 mg/m^2^/wk），阿霉素（40 mg/m^2^/wk）和依托泊苷（80 mg/m^2^×3 d/wk）；CEE方案：顺铂（75 mg/m^2^/3 wk），表柔比星（100 mg/m^2^/3 wk），依托泊苷（120 mg/m^2^×3 d/3 wk）；CAMP方案：CAP+泼尼松龙（1, 000 mg/m^2^×4 d和500 mg/m^2^×2 d/3 wk）。 T，胸腺瘤；TC，胸腺癌。 注：本表已获得版权所有者© 2011 by the International Association for the Study of Lung Cancer复制许可。											

### 1.2 化疗记录的要求

各研究中心报告术前、术后和姑息性化疗时，应包括下列内容：①应用何种细胞毒类药物；②化疗周期数；③最终是否实施>70%计划剂量^[22]^。此阈值常用于Ⅱ期临床试验，以确定化疗药物的有效性。应当根据NCI不良反应分级标准（CTC），系统地评估并记录急性和慢性化疗毒性反应^[23]^。记录3-5级毒性反应和剂量限制性毒性反应。胸腺瘤患者生存时间长于其他恶性肿瘤，因此研究者应额外关注慢性和迟发性毒性反应的准确评估。尤其值得注意的是，蒽环类药物与放疗或手术联用可增加心脏毒性，可引起副肿瘤性心肌炎^[24]^。

胸腺肿瘤化疗疗效评价应按ITMIG规定的标准描述^[25]^。胸腺瘤和胸腺癌的结果应分开报告，疗效评价的主要手段为CT扫描。接受奥曲肽治疗的患者，应记录治疗前奥曲肽扫描结果^[26]^。建议系统地记录抗肿瘤治疗及对副瘤综合征治疗的效果，如特异性的药物或免疫抑制剂的应用^[27]^。

### 1.3 根治性化疗

根治性化疗的模式包括术前或放疗前化疗、同步放化疗以及术后化疗（表 1和图 1）。

**1:**
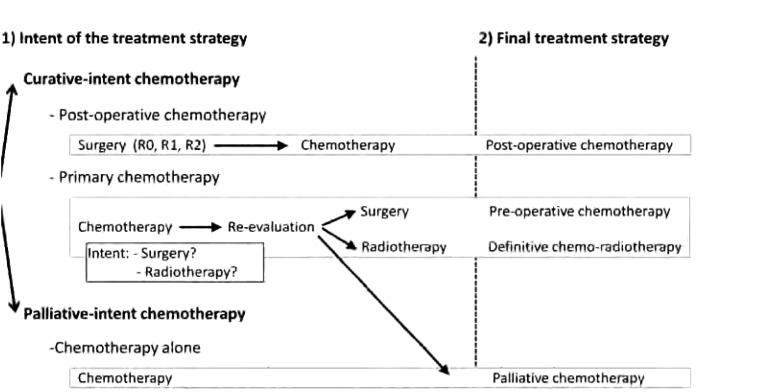
胸腺恶性肿瘤治疗方案及定义总结 Summary and defini-tions of treatment strategies involving chemotherapy in thymic malignancies

#### 1.3.1 初始化疗

初始化疗是指无远处转移的局部进展期胸腺肿瘤患者的初始治疗（Masaoka-Koga分期为Ⅱ期-Ⅵa期的患者）^[28]^。胸腺瘤对化疗的敏感为初始化疗提供了理论基础。初始化疗的主要目的是增加R0切除率，后者是胸腺肿瘤最重要的预后因素^[29]^，常用化疗药物见表 2。通常行2-4周期初始化疗，末次化疗后3-4周行增强CT评效。如果手术切除是局部进展期胸腺肿瘤的主要治疗目的，那么最终治疗方案取决于初始化疗后的肿瘤反应（图 1）。在最大宗的研究报道中，70%-80%患者可以获得缓解（表 2）。预期能够获得R0切除的患者接受手术。末次化疗至手术的间隔时间应小于8周。在最大宗的研究报道中，大部分患者在化疗后接受了手术，其中约50%的患者达到R0切除，对于这些患者的化疗则定义为“术前化疗+手术”。若患者不适合行手术治疗，如无法达到R0切除，或无法耐受手术者，可以在化疗后考虑根治性放疗（表 1）^[30]^。根治性放疗在无法行手术治疗的患者中的治疗意义需要进一步研究探索。文献报道，0-50%接受化疗的患者最终在化疗后接受了放疗，因此，对此类患者最终治疗则定义为“根治性放化疗”（表 2）。末次化疗至放疗的间隔时间应小于6周。化疗也可以和放疗同步进行。如果肿瘤负荷导致放疗剂量过大，或合并症导致放疗相关毒性风险增加，从而无法行放疗仅作了单一的化疗（图 1），则治疗策略就应定义为单纯化疗。单一化疗是姑息性治疗，文献报道0-21%局部进展期胸腺肿瘤患者接受初始化疗后，最终无法行手术、放疗或其他局部治疗而最终的治疗策略被定义为姑息性化疗（表 2）。

对局部进展期胸腺瘤化疗的报告建议（表 1）：①应该用“初始化疗”代替“诱导化疗或新辅助化疗”；②避免使用“可切除、潜在可切除、不可切除”等术语，而应提供肿瘤的整体分期^[28]^；③报告中应指出初始化疗目的，如术前或放疗前化疗；④应报告全部患者的最终治疗策略，包括术前化疗+手术、放疗前化疗+放疗、根治性化放疗、或姑息性化疗；⑤胸腺瘤和胸腺癌应单独分析。值得一提的是，一些研究中报告了局部进展期胸腺肿瘤患者术前化疗联合序贯或同步放疗，这样的治疗策略应定义为“术前放化疗”（表 2）。

#### 1.3.2 术后化疗

胸腺瘤根治性手术后复发率低，仅有少数文献报道术后化疗在胸腺瘤中的作用^[29]^。辅助化疗药物和初始化疗用药类似。法国一项研究^[31]^报道了21例Ⅲ-Ⅳa期的胸腺瘤患者，术后化疗方案为环磷酰胺+阿霉素+顺铂。MD Anderson肿瘤中心的两个术后放疗+化疗研究中，应用的方案为环磷酰胺+阿霉素+顺铂+泼尼松^[15, 32]^。目前的指南不推荐胸腺瘤患者术后化疗。

然而，胸腺癌患者常出现术后局部或远处复发^[33]^。在一项92例胸腺癌患者的报道中，术后化疗组5年生存率82%，术后放化疗组5年生存率47%，术后放疗组5年生存率74%，无术后治疗组5年生存率72%^[29]^。但该报道样本量较小，因此需要多中心合作，设计更好的研究进一步探索术后化疗的作用。

关于胸腺肿瘤术后化疗的报告，建议：①使用“术后化疗”代替“辅助化疗或巩固化疗”以使其可以名副其实并与放疗的表述一致^[30]^；②将胸腺瘤和胸腺癌单独分析；③应记录切缘状况（R0/R1/R2）。R2切除术后的化疗也定义为术后化疗，因为手术是根治性目的而并非诊断性目的，这点和术后放疗的定义一致^[30]^。R2切除术后，放疗可以和化疗序贯或同步应用，称之为术后放化疗。术后化疗应在术后12周内开始，若术后计划行放疗和化疗，放疗在12周内开始，化疗在12周后开始。术后检查发现复发或疾病进展后行化疗，应定义为“疾病复发化疗”，而并非术后化疗。

#### 1.3.3 姑息性化疗

姑息性化疗作为转移性胸腺肿瘤的基本治疗手段，通常不再进行手术或放疗（表 1）。局部进展期胸腺肿瘤）行化疗后无法行手术或放疗者，定义为姑息性化疗（图 1），其目的是改善肿瘤相关症状，期望获得肿瘤缓解和延长疾病控制，而并非根治肿瘤。已有数个前瞻性和回顾性研究报道了姑息性化疗应用的药物疗效，但均不是随机对照研究，无法判断哪种方案效果更好（表 3）。蒽环类药物为基础的化疗方案似乎缓解率较高。总之，推荐使用至少3种化疗药物且不多于6个周期的联合化疗方案。在姑息性化疗中，随着疾病进展可能需要使用多个化疗方案。推荐使用“一线化疗、二线化疗、三线化疗”这样的描述。

**3:** 晚期胸腺恶性肿瘤姑息化疗方案 Palliative chemotherapy regimens in advanced thymic malignancies

研究	例数	累计周期	组织类型	研究类型	方案	药物	剂量	反应率(%)
单药化疗								
Bonomi等^[20]^	21	4	T/TC	PhaseⅡ	Cisplatin		50 mg/m^2^/3wk	10-62
Highley等^[21]^	15	12	T/TC	Retrospective	Ifosfamide		1.5 g/m^2^×5 d/3wk	46-54
Loehrer等^[34]^	27	1	T/TC	PhaseⅡ	Pemetrexed		50 mg/m^2^/3wk	17
多药联合化疗								
Fornasiero等^[2]^	32	11	T	Retrospective	ADOC	Adriamycin	40 mg/m^2^/3wk	85-92
						Cisplatin	50 mg/m^2^/3wk	
						Vincristin	0.6 mg/m^2^/3wk	
						Cyclophosphamide	700 mg/m^2^/3wk	
Loehrer等^[10]^	30	9	T/TC	PhaseⅡ	CAP	Cisplatin	50 mg/m^2^/3wk	51
						Adriamycin	50 mg/m^2^/3wk	
						Cyclophosphamide	500 mg/m^2^/3wk	
Giaccone等^[11]^	16	6	T	PhaseⅡ	PE	Cisplatin	60 mg/m^2^/3wk	56-60
						Etoposide	120mg/m^2^×3/wk	
Loehrer等^[12]^	34	2	T/TC	PhaseⅡ	VIP	Etoposide	75 mg/m^2^×4 d/wk	32
						Ifosfamide	1.2 g/m^2^×4 d/wk	
						Cisplatin	20 mg/m^2^×4 d/wk	
Lemma等^[13]^	46	7	T/TC	PhaseⅡ	Carbo-Px	Carboplatin	Area under the curve	33
						Paclitaxel	5/3wk 225mg/m^2^/3wk	
T，胸腺瘤；TC，胸腺癌。 注：本表已获得版权所有者© 2011 by the International Association for the Study of Lung Cancer复制许可。								

#### 1.3.4 复发后化疗

复发后化疗是指接受根治性治疗，肿瘤完全消失后出现肿瘤复发而给予的化疗。姑息性化疗后未获得完全根治的患者进展后所行的再次化疗，应定义为二线或三线化疗，而不是复发后化疗。与上述情况类似的是复发性胸腺肿瘤治疗也可能是根治性的，治疗方式包括化疗、手术、放疗。对于放疗，建议使用和初始化疗相同的术语，但要指出是复发后放疗。

## 2 皮质醇在胸腺肿瘤中的应用

### 2.1 对疗效评估的影响

已知皮质醇有减少淋巴细胞的效应^[35, 36]^。和未接受皮质醇治疗的患者相比，接受皮质醇治疗患者的胸腺脂肪、纤维结缔组织含量明显增加，生发中心减少，皮髓质分化更差。皮质醇治疗后的改变类似于老龄或急性应激反应，保留肌上皮基质，但胸腺淋巴组织减少。在淋巴细胞型胸腺瘤（AB/B1/B2）中，通过减少淋巴细胞，皮质醇可使病灶明显缩小，但并无抗肿瘤效应。目前有关皮质醇在胸腺瘤治疗中作用的研究数据较少。ECOG（东部肿瘤协作组）的一项Ⅱ期临床试验对比了联用奥曲肽和皮质醇（泼尼松0.6 mg/kg/d）和单用奥曲肽的疗效，接受皮质醇治疗组患者缓解率更高（38%与11%）^[26]^。

既然皮质醇对疗效评估有一定影响，因此建议在研究中报告患者是否接受大于0.5 mg/kg/d的泼尼松或等效剂量的其他类固醇激素，以及皮质醇治疗的时间。0.5 mg/ kg/d泼尼松的阈值来源于ECOG临床试验中的数据，借此区分于其他为减低化疗反应而临时使用的低剂量激素。建议单独报告和分析接受皮质醇治疗和未接受皮质醇治疗两组患者的反应率。皮质醇治疗后含淋巴细胞的胸腺瘤的疗效评价标准是否应该不同于不含淋巴细胞的胸腺瘤尚不清楚，需要前瞻性研究证实。

### 2.2 对复发/进展定义的影响

已知感染、烧伤、皮质醇治疗或化疗停止后，尤其是婴幼儿应用高剂量化疗后，均可引起胸腺组织增大，此现象称之为胸腺反跳性增生^[37-39]^。据报道反跳性增生可发生于皮质醇停药后2个月-14个月，持续2个月-45个月，胸腺组织体积增大，淋巴滤泡数量不增加^[40]^。对于胸腺瘤患者尚未报道反跳性增生，但需要讨论其对疗效评估的影响。建议对停止化疗或皮质醇治疗后15个月内表现为局部复发的患者要考虑反跳性增生的可能。

特殊的影像学表现可以帮助鉴别胸腺增生和肿瘤复发，典型胸腺增生表现为胸腺组织对称性、均匀性增大。胸腺增生和胸腺肿瘤均可能与血管关系密切但未达到侵犯的程度^[40]^。FDG标记的PET扫描在鉴别诊断上没有帮助，增生和肿瘤表现出相似的葡萄糖摄取率^[41]^。有探索增强MRI在鉴别胸腺瘤和胸腺增生方面作用的研究^[42]^。治疗结束后15个月内发生的胸腺增大，如果通过影像检查无法确诊，则推荐活检。

总之，化疗在胸腺肿瘤中的作用尚待进一步研究，已有的材料有许多定义上的混乱，本文就胸腺瘤化疗的概念作了介绍，希望有助于前瞻数据库的建立。
